# Prevention And Treatment of Hypertension With Algorithm-based therapy (PATHWAY) number 2: protocol for a randomised crossover trial to determine optimal treatment for drug-resistant hypertension

**DOI:** 10.1136/bmjopen-2015-008951

**Published:** 2015-08-07

**Authors:** Bryan Williams, Thomas M MacDonald, Mark Caulfield, J Kennedy Cruickshank, Gordon McInnes, Peter Sever, David J Webb, Jackie Salsbury, Steve Morant, Ian Ford, Morris J Brown

**Affiliations:** 1 Institute of Cardiovascular Sciences University College London (UCL) and National Institute for Health Research (NIHR) UCL/UCL Hospitals Biomedical Research Centre, London, UK; 2 Medicines Monitoring Unit, Medical Research Institute, University of Dundee, Dundee, Tayside, UK; 3 William Harvey Institute, QMUL, London, UK; 4 Cardiovascular Medicine & Diabetes, King's College London, London, UK; 5 Institute of Cardiovascular Medical Sciences, Western Infirmary, University of Glasgow, Glasgow, UK; 6 Centre of Circulatory Health, Imperial College, London, UK; 7 Clinical Pharmacology Unit, University of Edinburgh, Edinburgh, UK; 8 Clinical Pharmacology Unit, Addenbrooke's Hospital, University of Cambridge, Cambridge, UK; 9 Robertson Centre, University of Glasgow, Glasgow, UK

**Keywords:** Resistant Hypertension, Drug Treatment

## Abstract

**Introduction:**

Resistant hypertension is inadequately controlled blood pressure (BP) despite treatment with at least three BP-lowering drugs. A popular hypothesis is that resistant hypertension is due to excessive Na^+^-retention, and that ‘further diuretic therapy’ will be superior to alternative add-on drugs.

**Methods and analysis:**

Placebo-controlled, random crossover study of fourth-line treatment when added to standard (A+C+D) triple drug therapy: ACE inhibitor or Angiotensin receptor blocker (A) +Calcium channel blocker (C)+Diuretic (D). Patients (aged 18–79 years) with clinical systolic BP≥140 mm Hg (135 mm Hg in diabetics) and Home BP Monitoring (HBPM) systolic BP average ≥130 mm Hg on treatment for at least 3 months with maximum tolerated doses of A+C+D are randomised to four consecutive randomly allocated 12-week treatment cycles with an α-blocker, β-blocker, spironolactone and placebo. The hierarchical coprimary end point is the difference in HBPM average systolic BP between (in order) spironolactone and placebo, spironolactone and the average of the other two active drugs, spironolactone and each of the other two drugs. A key secondary outcome is to determine whether plasma renin predicts the BP response to the different drugs. A sample size of 346 (allowing 15% dropouts) will confer 90% power to detect a 3 mm Hg HBPM average systolic BP difference between any two drugs. The study can also detect a 6 mm Hg difference in HBPM average systolic BP between each patient's best and second-best drug predicted by tertile of plasma renin.

**Ethics and dissemination:**

The study was initiated in May 2009 and results are expected in 2015. These will provide RCT evidence to support future guideline recommendations for optimal drug treatment of resistant hypertension.

**Trial registration number:**

Clinicaltrials.gov NCT02369081, EUDract number: 2008-007149-30.

**Strengths and limitations of this study**

- Randomised, masked, adequately powered study of rigorously defined resistant hypertension;
- Rigorously characterised population of patients with resistant hypertension.
- Use of home blood pressure monitoring to evaluate the blood pressure response to treatment.
- The trial is not powered to detect the impact of drug treatment on morbidity and mortality.
- Spironolactone is not licensed for the treatment of hypertension in UK.

## Introduction

Many patients treated for hypertension do not achieve their target blood pressure, defined according to most guidelines as a seated clinic blood pressure (BP) <140/90 mm Hg. There are various reasons for poor BP control, including clinical inertia in up-titrating therapy and poor patient concordance with treatment. There are also patients who appear to be genuinely resistant to drug treatment, hence the term ‘resistant hypertension’.^1^ Resistant hypertension has been defined in various ways, but recent international guidelines have moved towards a consensus that resistant hypertension is best defined as poor BP control despite treatment with at least three BP-lowering medications, one of which should be a diuretic.^2^ ^3^ The recent National Institute for Health and Care Excellence (NICE) guideline was even more prescriptive by defining resistant hypertension as a blood pressure that is not controlled to recommended treatment targets despite treatment with maximal recommended or best tolerated doses of three specific drug types, that is, A+C+D, according to the NICE hypertension treatment algorithm, where ‘A’ is an **A**CE-inhibitor or **A**ngiotensin receptor blocker (ARB), ‘C’ is a **C**alcium channel blocker (CCB) and ‘D’ is a thiazide/thiazide type diuretic.^4^ ^5^ Thus, when referring to the drug treatment of resistant hypertension, guidelines are specifically referring to fourth-line drug treatment, usually added to treatment with A+C+D.

With regard to drug treatment of hypertension, a ‘rule of thirds’ seems to apply, with approximately one-third of hypertensive patients achieving BP control with a single drug, one-third requiring two drugs and one-third requiring three drugs or more. Some of those requiring three drugs or more will be controlled with three drugs, with most surveys suggesting that approximately 10% of patients will require more than three drugs to control BP, and thus are defined as patients with resistant hypertension.^1^ ^6^ The true prevalence of resistant hypertension has been poorly defined, but based on the aforementioned assumptions the prevalence in the UK alone would exceed 1 million people.

The choice of fourth-line drug treatment for resistant hypertension has been entirely empirical. This reflects the lack of data from randomised controlled trials directly comparing different drug treatment options for resistant hypertension; consequently, there remains real clinical uncertainty about the preferred clinical management of such patients. It is possible that it makes no difference what drug is added as fourth-line treatment and that the response, on average, will be the same for all available treatment options. Alternatively, it is possible that one class of drug will usually be superior to all the others because there is a common mechanism underpinning resistant hypertension for the majority of patients. In this regard, a popular view is that resistant hypertension is predominantly due to excessive sodium retention and thus ‘further diuretic therapy’ will usually be the most effective treatment.^3^ ^7^ A third possibility is that resistant hypertension is a more heterogeneous state and that the study of average responses in cohorts in clinical studies masks substantial individual patient differences and that treatment decisions could potentially be better stratified.

With regard to treatment stratification, one approach would be to use biomarkers of the patient's sodium/volume status, the rational being that an excess of total body sodium would contribute to treatment resistance and could help define the best treatment for patients with resistant hypertension. In this regard, plasma renin level is a surrogate for and inversely related to sodium status.^8^ This may be especially true in patients already receiving treatment with A+C+D, all of which would be expected to increase plasma renin levels. In this setting, we hypothesised that the finding of low plasma renin (lowest tertile) would be strongly suggestive of excessive sodium load as a mechanism for resistant hypertension and thus, might predict that the best treatment response would be obtained with further diuretic therapy as the preferred fourth-line treatment. Conversely, the finding of an elevated plasma renin (highest tertile) would point to a better response to a drug that suppresses plasma renin, that is, a β blocker, whereas those in the mid-tertile of plasma renin would respond best to a α-blocker as fourth-line treatment.

We selected spironolactone as the ‘further diuretic’ therapy to be tested because of observational data suggesting its efficacy in lowering BP in resistant hypertension.^9–11^ However, the downside with spironolactone is the development of gynaecomastia in some patients with prolonged usage.^9^ A previous crossover comparison of diuretics showed that a potential alternative to spironolactone in this setting is amiloride.^12^ Thus, PATHWAY-2 will obtain prospective, sequential comparative evidence for amiloride in resistant hypertension by offering this treatment as an open-label run-out to all patients after completion of the prospective randomised treatment cycles, at doses likely to have comparable efficacy to the doses of spironolactone. The PATHWAY 2 study was initiated in May 2009, and the final results are expected in 2015. This manuscript describes the objectives, design and respecified analysis plan for the PATHWAY 2 study.

## Methods

### Study design

PATHWAY 2 is a randomised, double blind, placebo-controlled, crossover, multicentre trial comparing the four treatment options for patients with resistant hypertension. The four treatment options are taken once daily, in addition to the maximum/best tolerated doses of three drugs at baseline according to the current NICE hypertension treatment guideline, that is, A+C+D where: A=ACE-inhibitor or Angiotensin receptor blocker; C=Calcium channel blocker; D=thiazide/thiazide-type Diuretic.^4^ ^5^

**Figure 1:**
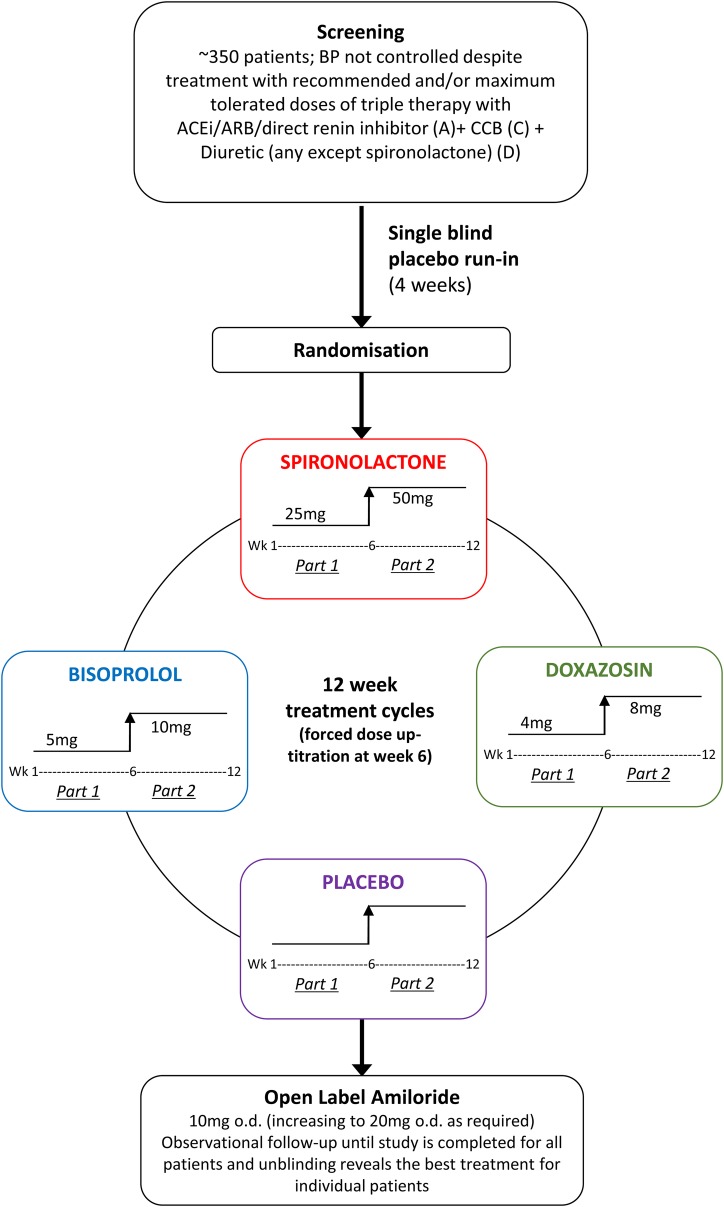
PATHWAY 2 study design and flow chart.

After a screening visit, patients potentially eligible for randomisation proceeded to a 4-week run-in period on the patient's baseline therapy with three drugs, that is, A+C+D. After the run-in period and if still meeting the eligibility criteria, the patients were allocated double blind to a cycle of 4×once daily oral treatments in a random sequence. The randomisation is not stratified and in this 4 × 12-week period, four treatments crossover study, participants are randomised with equal probability to one of the 24 possible sequences of treatments. The treatments comprise: (1) further diuretic therapy—spironolactone; 25–50 mg, (2) α-blocker—doxazosin modified release; 4–8 mg, (3) β-blocker—bisoprolol; 5–10 mg and (4) placebo. The treatment cycles were initiated with each treatment for 6 weeks at the lower dose, followed by forced titration to twice this dose for a further 6 weeks—each cycle for a total of 12 weeks. There is no washout period between the four treatment cycles (3 active, 1 placebo), as 12 weeks of treatment per drug cycle is considered sufficient to eliminate the order effects of treatment. The total length of the trial is 52 weeks (4 weeks run-in and 4×12 week treatment cycles; figure 1).

The trial was designed as double-blind, but this incurs considerable extra costs; thus, we decided that the last 100 patients should be studied open-label, in order to determine whether the large extra cost of blinding confers any advantage. For the blinded phase of the trial, the study drugs will be re-encapsulated to provide participants with an identical single capsule throughout, containing either active drug or placebo (microcrystalline cellulose). For the open label cohort, patients will receive drugs according to the randomisation schedule in the original drug manufacturers’ packaging.

During the double-blind phase, none of the investigators, patients or laboratory staff were aware of the assignment. A 24-h telephone unblinding service was provided by the Data Management Centre for instances where principal investigators (PIs) believed that treatment of an adverse event may be compromised by their not knowing the treatment assignment.

Compliance has been assessed by returned tablet counts.

#### Tolerability

Adverse events are recorded in the electronic case record form at each visit. A 2-week drug holiday is permitted at any point if the investigator considers that this may allow participants to remain in the trial without early withdrawal.

#### Run-out phase of the study

To evaluate if amiloride would be a suitable alternative to spironolactone, the run-out phase of the study was used to evaluate the BP-lowering effectiveness of amiloride using an open label design. Patients were eligible for the amiloride open label run-out phase of the study as long as they did not develop potassium levels ≥5.5 mmol/L during the study treatment cycles. If eligible, patients were prescribed open-label amiloride 10 mg once daily for 6 weeks, followed by forced titration to amiloride 20 mg once daily for a further 6 weeks. Patients were evaluated at study sites at 6 and 12 weeks, and BP recorded in accordance with procedures for the main study. In the remaining patients, physicians will choose the exit drug based where possible on pretrial experience of fourth and fifth-line treatment. For patients with uncontrolled BP at the end of the treatment cycles, or the amiloride run-out phase, it was considered unethical to wait for completion of the overall study before their best treatment option could be revealed. Thus, at the end of the main study, the data base will be locked for individual patients and the data centre can reveal the best treatment option for an individual patient at the request of the study site PI. The full schedule of assessment and procedures is shown in the table 1.

**Table 1:** PATHWAY 2 Study schedule and procedures

	Screening	Randomisation	Phase 1		Phase 2		Phase 3		Phase 4	
	Week −4	Week 0	Week 6	Week 12	Week 18	Week 24	Week 30	Week 36	Week 42	Week 48
Informed consent	X									
Medical history	X	X								X
Clinical examination	X									X
Concomitant medications	X	X	X	X	X	X	X	X	X	X
Weight	X	X	X	X	X	X	X	X	X	X
Inclusion/exclusion criteria check	X	X								
Clinic BP and heart rate	X	X	X	X	X	X	X	X	X	X
Home BP and heart rate*		X	X	X	X	X	X	X	X	X
Home BP 6 h after witnessed administration of A+C+D medication†		X								
12 lead ECG	X									X
PEFR	X	X	X	X	X	X	X	X	X	X
Blood tests—electrolytes, urea, creatinine	X	X	X	X	X	X	X	X	X	X
Blood tests—glucose, lipids, uric acid, Ca++	X									X
Blood tests—cholesterol, HDL and triglycerides		X								X
Blood tests –plasma renin/aldosterone‡		X								
Pharmacogenetics§		X								
Blood tests—haematology, albumin, glucose	X			X		X		X		X
Blood—serum ace levels		X								
24 h urinary electrolytes		X								
Urinalysis	X	X		X		X		X		X
HCG testing pregnancy	X	X		X		X		X		
Haemodynamic measures (cardiac output, peripheral resistance, bioimpedance), pulse wave analysis/velocity¶		X		X		X		X		X
Compliance check		X	X	X	X	X	X	X	X	X
Directly observed therapy		X								
AE reporting			X	X	X	X	X	X	X	X
Randomisation		X								
Dispensing	X	X	X	X	X	X	X	X	X	X

*Home BP measurements are recorded twice daily in triplicate in the 4 days leading up to the baseline visit and week 6 and week 12 visits of each treatment cycle.

†To exclude white coat hypertension and confirm adherence.

‡In the event that plasma renin/aldosterone is not taken at baseline, it will be measured at week 12 of each treatment cycle.

§Pharmacogenetics sample to be taken where specific informed consent has been given. Sampling will typically be at baseline (day 0) but may be at any time later in the study.

¶Where equipment is available.

AE, adverse events; BP, blood pressure; HCG, human chorionic gonadotropin; HDL, high-density lipoprotein; PEFR, peak expiratory flow rate.

#### Special considerations

Early randomisation during the 4-week placebo run-in phase, that is, 2 weeks, is allowed for safety reasons for patients developing an excessively elevated BP >180/110 mm Hg or if a fourth background drug has been discontinued at the screening visit or at the discretion of the study site PI. A preplanned break of up to 3 months from the study is permitted, providing patients have completed the previous treatment cycle. A 2-week break from study drugs is permitted to accommodate patients with scheduling problems or for patients with adverse events not definitely attributable to the study drugs. Patients unable to tolerate a drug in a cycle are permitted to move to the next drug in sequence. Extra study visits after dose titration may be arranged at the discretion of the study site PI.

#### Data handling and recording

Study data is recorded via remote data entry into a web-based electronic case report form (eCRF) developed for the study. The data will be collated and held at a single study data management centre, the Robertson Centre for Biostatistics (RCB) and Glasgow Clinical Trials Unit (CTU). The eCRF data is anonymous and will identify study participant by their assigned study numbers only.

#### Ethics approval and study registration

The PATHWAY 2 study has been approved by all relevant ethics committees and all participants will sign informed consent. Consent procedures will be undertaken by trained nursing or clinical staff with delegated authority of the local PI. The PATHWAY 2 study is registered with clinicaltrials.gov; trial identifier; NCT02369081 and EUDract number: 2008-007149-30.

The initial protocol was approved by the Medicines and Healthcare Products Regulatory Agency (MHRA) on 14 January 2009, and is visible at https://www.clinicaltrialsregister.eu/ctr-search/trial/2008-007149-30/GB. It was approved by Cambridge Research Ethics Committee on 12 January 2009. The trial was not registered with Clinicaltrials.gov until 2015 because of the prior registrations with MHRA and UKCRN, and local advice that these sufficed. The current version is 9, dated 23 August 2012. Any further amendments will be approved by Research Ethics and MHRA and also registered with clinicaltrials.gov.’

#### Study participants

The study will randomise 346 patients aged 18–79 years from 14 study sites in the UK. These are the sites represented by the authors, plus Birmingham, Norwich, Manchester, Ixworth and Exeter. Full addresses are at clinicaltrials.gov. All patients will have uncontrolled BP, that is, resistant hypertension despite treatment with maximal/best tolerated doses of a combination of three drugs, that is, A+C+D, according to the recommendations of the current NICE hypertension treatment guidelines.^4^ ^5^ Uncontrolled BP is defined as seated clinic systolic BP ≥140 mm Hg for patients without diabetes or ≥135 mm Hg for patients with diabetes, based on a mean of the last 2 of 3 consecutive clinic readings. In addition, the HBPM systolic BP average must be ≥130 mm Hg and within 15 mm Hg of the clinic systolic BP, in the 4 days prior to the baseline visit. Patients who have received three drugs, that is, A+C+D, but are either intolerant to one drug class or tolerate only a lower dose, that is, submaximal recommended dose are eligible. Patients receiving A+C+D and who are also receiving additional drugs for their hypertension may also be included if the investigator (1) considers it is safe and appropriate to stop these additional drug/s at the screening visit and (2) anticipates that the BP criteria for inclusion will still be met after discontinuing the drug/s when rechecked at the baseline visit. Patients can also be included if the HBPM average systolic BP exceeds 130 mm Hg during the run-in period.

Patients are excluded if their clinic systolic BP is >200 mm Hg or diastolic BP >120 mm Hg, but the PI has discretion to over-ride this criterion if home BP measurements are lower. Patients were also excluded if they had secondary or accelerated hypertension; type 1 diabetes (but not type 2 diabetes); an eGFR<45 mL/min or a plasma potassium outside of normal range on two successive measurements during screening, pregnancy, while planning to conceive, or were women of childbearing potential, that is, not using effective contraception; had an anticipated change of medical status during the trial (eg, surgical intervention requiring >2 weeks convalescence); had an absolute contraindication to any of the study drugs (eg, asthma) or a requirement for study drug for reasons other than to treat hypertension (eg, β-blockers for angina or diuretics other than those to treat hypertension, or previous intolerance of the proposed trial therapies, or had sustained atrial fibrillation or a recent (<6 months) cardiovascular event requiring hospitalisation (eg, myocardial infarction or stroke)). Patients were excluded if they were suspected of non-adherence to antihypertensive treatment as determined by a pill count at the end of the 4- week run-in period—those with adherence <70% will be excluded from randomisation, and those with a fall in home systolic BP to <130 mm Hg, 6 h after witnessed administration of their usual A+C+D therapy. A full list of inclusion and exclusion criteria are shown in boxes 1 and 2.

Box 1 **PATHWAY 2 study inclusion criteria**

1. Patients aged 18–79 years
2. Patients will all have hypertension that is not controlled to target: clinic systolic BP ≥5 mm Hg above target (ie, ≥145 mm Hg for non-diabetic hypertensives or ≥135 mm Hg for patients with diabetes), under one of the following conditions:
  - Treatment for at least 3 months with lisinopril 20 mg (A)+amlodipine 10 mg (C)+bendroflumethiazide 2.5 mg (D) or their equivalents
  - Patients who have received the three drugs or equivalents specified in (a), and are either intolerant to one category, or tolerate only a lower dose (eg, amlodipine 5 mg or lisinopril 10 mg)
  - Patients receiving the three drugs or equivalents specified in (a), who are receiving additional drugs for their hypertension, may be included if the investigator: (1) feels it is appropriate to stop these additional drugs at the screening visit and (2) anticipates that the BP criteria for inclusion will be met when rechecked at the baseline visit
3. Patients with a home systolic BP average of >130 mm Hg or within 15 mm Hg of clinic BP over the 4 days prior to the baseline visit.

Box 2 **PATHWAY 2 study exclusion criteria**

Inability to give informed consent;

Participation in a clinical study involving an investigational drug or device within 4 weeks of screening;

Secondary or accelerated hypertension;

Type 1 diabetes;

eGFR<45 mL/min;

Plasma potassium outside of normal range on two successive measurements during screening;

Pregnancy, planning to conceive or women of childbearing potential, that is, not using effective contraception;

Anticipated change of medical status during the trial (eg, planned surgical intervention requiring >2 weeks convalescence);

Absolute contra-indication to study drugs (eg, asthma) or previous intolerance of trial therapy;

Sustained atrial fibrillation;

Recent (<6 months) cardiovascular event requiring hospitalisation (eg, myocardial infarction or stroke);

Suspected non-adherence to antihypertensive treatment (see above);

Requirement for study drug for reason other than to treat hypertension, (eg, β-blockers for angina or diuretics other than those to treat hypertension);

Current therapy for cancer;

Concurrent chronic illness, or other reasons likely to preclude 52 week participation in the study;

Clinic Systolic BP >200 mm Hg or diastolic BP >120 mm Hg, with PI discretion to override if home BP measurements are lower

Any concomitant condition that, in the opinion of the investigator, may adversely affect the safety and/or efficacy of the study drug or severely limit that patients life-span or ability to complete the study (eg, alcohol or drug abuse, disabling or terminal illness, mental disorders);

Treatment with any of the following medications;

- Oral corticosteroids within 3 months of screening. Treatment with systemic corticosteroids is also prohibited during study participation;
- Chronic stable use, or unstable use of NSAIDs (other than low dose aspirin) is prohibited. Chronic use is defined as >3 consecutive or non-consecutive days of treatment per week. In addition intermittent use of NSAIDs is strongly discouraged throughout the study and NSAIDs if required, must not be used for more than a total of 2 days. For those requiring analgesics during the study, paracetamol is recommended.
- The use of short-acting nitrates (eg, sublingual nitroglycerin) is permitted. However, participants should not take short acting oral nitrates within 4 h of screening or an subsequent visit;
- The use of long-acting nitrates (eg, Isordil) is permitted but the dose must be stable for at least 2 weeks prior to screening and randomisation;
- The use of sympathomimetic decongestants is permitted, however, not within 1 day prior to any study visit/BP assessment;
- The use of theophylline is permitted but the dose must be stable for at least 4 weeks prior to screening and throughout the study;
- The use of phosphodiesterase type V inhibitors is permitted; however study participants must refrain from taking these medications for at least 1 day prior to screening or any subsequent study visits;
- The use of α-blockers is not permitted, with the exception of afluzosin and tamsulosin for prostatic symptoms

A pill count will be made at the end of the 4 week run-in period and those with adherence <70% will be excluded from randomisation

#### Recruitment and randomisation of participants

Potentially suitable patients are identified from hospital and general practice populations. Written informed consent is obtained from participants by a medical investigator. The research nurse records baseline variables, takes blood and urine for baseline biochemistry and haematology, and records the medical history. Blood samples are analysed at the local health service laboratory according to usual practice. Serum for future analyses and blood for future genetic analyses are stored by centres. Participants who have given informed consent and meet the inclusion and exclusion criteria at the end of a month's placebo run-in are randomised to receive one of the possible sequences of the four trial treatments, each in addition to any other antihypertensive drug being taken at the time of randomisation. This is performed by contacting a central computerised randomisation facility based at the Robertson Centre for Biostatistics, University of Glasgow, by telephone or via a web-based service.

### Study objectives and end-points

#### Primary objective

The primary study objective is to test the hypothesis that the commonest cause of resistant hypertension is excessive sodium retention, and that ‘further diuretic therapy’, that is, spironolactone will be superior to other potential ‘add-on drugs’ for people with resistant hypertension, that is, inadequate BP control despite treatment with maximal/best tolerated doses of the three drug classes A+C+D, as recommended by the current NICE treatment guidelines algorithm.

#### Secondary objectives

The main secondary objective is test the use of plasma renin measurement to evaluate the ‘α, β, Δ’ rule^8^ for the selection of the fourth-line drug for patients with drug resistant hypertension—where α represents α-blockade, β represents β-blockade and **Δ** represents ‘further diuretic therapy’. We propose that α-blockade will be the most effective fouth-line treatment at lowering BP in patients in the mid-tertile of plasma renin (expected to be ≥20 mU/L but <100 mU/L); β-blockade will be the most effective drug when the renin level in in the top tertile (expected to be ≥100 mU/L) as this drug inhibits renin secretion; further diuretic therapy with spironolactone will be the most effective drug in the lowest tertile of plasma renin (<20 mU/L), indicative of renin suppression due to excessive sodium retention. Further secondary analyses will use physiological phenotyping of non-invasive haemodynamic parameters indicative of sodium retention and volume status, that is, cardiac output, peripheral vascular resistance, bio-impedance, pulse wave analysis and pulse wave velocity to evaluate mechanisms for resistant hypertension and determine if these methods can help better stratify the best treatment for individual patients with resistant hypertension.

### Study procedures

#### Clinic blood pressure measurement

Seated clinic blood pressure will be measured at each clinic visit in accordance with the methods recommended by the British Hypertension Society,^13^ using an automated, approved and validated BP monitor (Microlife Watch BP, Microlife) with an appropriate cuff size applied to the non-dominant arm. Three measurements will be recorded 1 min apart, after 5 min rest and seated, and the average of the last 2 measurements will be recorded as the clinic BP.

#### Home blood pressure monitoring

The HBPM systolic BP average will be used for the main study end points. At several points during the trial, HBPM readings will be recorded (immediately prior to randomisation and at the end of 6 and 12 weeks of each treatment cycle). BP will be measured using the non-dominant arm, using the same BP monitor used for the clinic BP measurement which will be issued to the patient for use throughout the trial. BP readings will be taken in the morning and in the evening, ideally at the same times throughout the study, for example, ∼08:00 and 20:00—or the nearest time to these that is convenient for the patient. HBPM readings will be taken on four consecutive days prior to a study visit. Participants will be asked to make triplicate readings, 1 min apart, after 5 min seated rest, and to record these on the pro forma provided. All readings will also be captured automatically by the monitor. For analysis, a maximum of the last 18 recordings (ie, days 2–4, if all completed) will be used. If the patient fails to comply with the recommended number of recordings, a minimum of six BP recordings is required for a valid measurement of the HBPM average.

#### Plasma renin measurement

Plasma renin levels will be measured using a Diasorin Liaison automated chemiluminescent immunoassay for direct renin*.* Performed in Cambridge (English centres) and Edinburgh (Scottish centres).

#### Assessment of drug treatment compliance

It is recognised that poor concordance with drug treatment contributes to apparent drug treatment resistance. Robust checks for treatment concordance are included in the study procedures: (1) At baseline and as a prerequisite for randomisation, patients will be witnessed consuming their usual BP medications (A+C+D) in the clinic and then HBPM will be performed over the next 6 h to confirm that their BP is not controlled with their usual treatment; (2) Tablet counts will be performed at baseline (end of placebo run-in) and after 12 weeks of each treatment cycle, and patients with less than 70% tablet consumption will be deemed non-concordant with their medication; (3) Serum ACE levels will be measured to provide a retrospective measure of treatment concordance; (4) Urine samples will be stored frozen at −20°C at selected sites throughout the study, for analysis of urinary drug metabolites at a single site (University of Leicester) using high-performance liquid chromatography/mass spectroscopy to provide a retrospective analysis of treatment concordance with individual drugs.^14^

#### Non-invasive phenotypic profiling of haemodynamics and volume status

Non-invasive haemodynamic measures (cardiac output, peripheral resistance, bioimpedance) using thoracic bioimpedance cardiography (Cardiodynamics USA) and pulse wave analysis and pulse wave velocity measurements (Sphymocor, Australia, or equivalent) will be recorded at baseline and at the end of each treatment cycle at selected study sites.

#### Pharmacogenetics

Blood samples will be taken at baseline and the DNA extracted and stored for pharmacogenetic analysis. At the end of the study, these samples will be transferred from each site to Cambridge.

#### Safety assessments

All observed or volunteered adverse events considered related to treatment will be recorded on the adverse events section of the eCRF. Study staff will pursue and obtain information to confirm whether the event meets the criteria for classification as a serious adverse event. An event will be deemed serious if it results in death, is life-threatening, requires hospitalisation, results in persistent or significant disability/incapacity, is a congenital anomaly or birth defect, or is another medically important event. Adverse events will be classified according to seriousness, severity (mild, moderate or severe), causal relationship (certain, probably, possible, unlikely, unrelated) and expectedness. Suspected unexpected serious adverse drug reactions (SUSAR's) are not considered likely in this trial as there have been many years of experience with each of the trial drugs. All potential SUSAR's are subject to expedited reporting. Evaluations of blood pressure, blood counts, blood chemistry, urinalysis, ECGs, physical examination and monitoring of vital signs are scheduled to be performed at regular intervals and can be increased in frequency if required, at the discretion of the PI.

#### Data handling and record keeping

Study data is recorded by remote data entry into a web-based electronic case report form (eCRF) developed for the study by the Robertson Centre, Glasgow. eCRF data is anonymous and will identify study participants by their assigned study numbers only. All missing data, possible duplication and data outside preset limits for each parameter is queried by the Management Centre, and will be internally validated before database lock.

### Statistical analysis plan

The study has a hierarchical coprimary end point which will be the difference in the home systolic BP between spironolactone and placebo, followed by the difference in home systolic BP between spironolactone and the average of the other two active drugs, (doxazosin and bisoprolol), followed by the difference in home systolic BP between spironolactone and each of the other two active drugs. For secondary analyses, similar analyses of the primary analysis will be made using clinic blood pressure. We will also compare the proportion of patients achieving target blood pressure (home and/or clinic) with each of the three active treatments. We will examine the proportion of patients controlled by each drug who are also controlled by the run-out treatment, amiloride. We will look at the direction of correlation between plasma renin and the blood pressure response to each drug. We will then describe differences between blood pressure response to each drug at extremes of plasma renin. Similar analyses will be performed for each haemodynamic variable. We will also examine the predictive value of the baseline measurements of cardiac output, systemic vascular resistance and thoracic impedance. The study will also explore the cost-benefit of using plasma renin to stratify best treatment in individual patients. An additional end point will be analyses of relationships between genetic factors and pharmacodynamic responses.

#### Sample size calculation

The sample size was based on detecting a difference of 3 mm Hg (SD=12 mm Hg) in systolic blood pressure between each of the experimental drugs and the placebo treatment with 90% power, and using a single sample t test at the 0.003 significance level (0.01 level adjusted for 3 comparisons being made). Variances in these calculations were estimated from the within-subject (interdrug) variability observed in 90 patients participating in the Cambridge crossover studies of the major drug classes.

The estimated requirement was for 294 patients; so we set a recruitment target of 346 to allowing for a dropout rate of 15%. This number also provides similar power to detect a twofold increase in superiority through renin profiling, namely a 6 mm Hg difference in systolic BP between best and second-best drug within each quartile of plasma renin.

#### Statistical model

The protocol requires patients to take triplicate morning and evening HBPM BP readings for the 4 days before each study visit, a total of 24 readings per visit. We shall use the arithmetic mean of last 18 measurements prior to a study visit to calculate home blood pressure. If more than 18 measurements have been obtained, the earliest recorded readings will be discarded. Thus, for most patients we will use all the readings on days 1 to 3 prior to the visit, but if any of these are missing then readings from day 4 will be used instead. The minimum number of measurements required for a valid assessment of home blood pressure using home BPM is six. Means of the morning and evening readings will be used in all subsequent analysis.

We shall use a mixed model to analyse HBPM BP, with unstructured covariances for the repeated measures for a patient. The model will include terms for time of day (morning or evening), gender, age, height, weight and smoking history. We will also adjust for baseline BP. If any of the baseline BPs cannot be estimated, we will substitute the overall mean baseline value, set an indicator variable to denote an imputed value and include the indicator variable in the model. The model will also include terms for treatment and phase (to allow for period effects), and an indicator variable to identify the subgroup of patients whose crossover treatments were unblinded.

*The primary analysis* will use BPs recorded for the maximum tolerated dose of each drug. The first of the primary end points is the mean difference between spironolactone and placebo; the second is the mean difference between spironolactone and the average of other active treatments; and the third and fourth are the mean differences between spironolactone and each of the other active treatments taken individually.

*The secondary analysis* will estimate for each treatment: changes in home BP; clinic BP on maximum tolerated dose, and changes in clinic BP; responder rates; withdrawals due to adverse events; and haemodynamic variables and pulse wave analyses. We shall use mixed models to analyse the continuous variables and these will include baseline covariates.

A patient will be deemed a “responder’ if they meet any of these two conditions; (1) HBPM systolic BP (SBP) <135 mm Hg; (2) HBPM SBP has fallen ≥10 mm Hg since baseline. Responder rates will be analysed by logistic regression and will be adjusted for baseline covariates. Withdrawal rates will be analysed by logistic regression and will be adjusted for baseline covariates.

**Figure 2:**
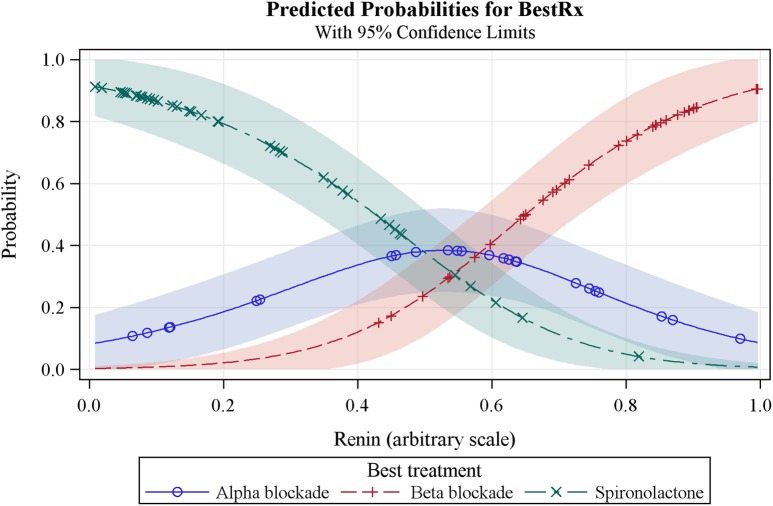
Predicted probabilities model for plasma renin as the predictor of the best treatment option for resistant hypertension.

The main secondary objective is to use plasma renin measurements to evaluate an ‘α, β, Δ’ rule for the selection of the fourth-line drug for patients with drug-resistant hypertension (see secondary objectives above). The hypothesis is that plasma renin (measured on a background of 3 drugs, ie, A+C+D), will predict the most effective fourth-line drug. We shall identify the best treatment for each patient, that is, the one on which they achieved the lowest BP. This is a categorical response and we shall use a logistic model to estimate the probability of each treatment being declared the best as a function of baseline renin. Superimposed plots of these probabilities (see figure 2) will show the range of renin values for which each treatment is most likely to be the best choice. We will also, in the same way, evaluate haemodynamic parameters indicative of sodium retention and volume status, that is, cardiac output, peripheral resistance and bioimpedance as predictors of the best fourth-line treatment among the three active treatments. An additional end point will be analyses of relationships between genetic factors and pharmacodynamic responses.

#### Blinded versus unblinded cohorts

We will assess the advantage and disadvantages of using the double-blind, placebo-controlled random crossover design in comparison to the open label random crossover design. We will expand the models used to analyse the primary and secondary outcomes to estimate a cohort effect (unblinded vs blinded) within each treatment.

Patients who withdraw from the study before final visit will be included in the primary analysis if they have at least one postrandomisation HBPM, and missing data imputed by application of the last observation carried forward. Patients with data missing from any time point required for analysis, and patients in whom major violation of the protocol is documented by investigators or detected by the data management centre will be excluded from per-protocol analysis.

There will be no interim analysis, no stopping rules and no data monitoring committee. This is because all treatments are being used for licensed indications, and have been so used for several decades. We do not, therefore, anticipate any unexpected hazard that has eluded detection during many millions of person-years exposure, and the study is not powered to detect any significant differences in serious morbidity or mortality between treatment groups.

### Ethics and dissemination

PATHWAY-2 is approved by Cambridge South Ethics Committee and the MHRA. The results will be published in a peer-reviewed journal, and presented at national and international meetings. All authors of this article will have full access to the complete data set, subject only to agreement by coauthors to uses of the data. Authorship of future articles reporting outcomes will represent multidisciplinary input at each site, with the articles being written by a subset of the current authorship. There are no current plans to make anonymised participant-level data publicly available. However, lay-friendly summaries of our findings will be sent to all our patients, and we expect to work with the British Heart Foundation to maximise patient and public access to the findings.

### Ancillary and post-trial care

During the trial, all patients are covered by the NHS indemnity. We expect most patients to continue the most effective trial treatment, in addition to other pretrial background therapy that has been continued during the trial.

### Study sponsorship: monitoring, audit, quality control and quality assurance

The trial is sponsored by the University of Cambridge and Cambridge University Hospitals NHS Foundation Trust, contact stephen.kelleher@addenbrookes.nhs.uk. Trial investigators will permit authorised third parties access to the trial site and medical records relating to trial participants. This will include, but not necessarily be restricted to, access for trial-related monitoring, audits, Ethics Committee review and regulatory inspections. We do not expect funders or sponsors to be involved in data analysis or reporting.

### Associated projects

This study (PATHWAY-2) is one of three complementary studies in a BHF-funded programme which will investigate optimal treatment for patients with hypertension. PATHWAY-1^15^ will investigate whether initial treatment with a combination of drugs is more effective in achieving a sustained target pressure than starting with monotherapy and adding a second drug. PATHWAY-3^16^ will assess the impact of thiazide diuretic versus a combination of thiazide diuretic and potassium-sparing diuretic on glucose tolerance and BP lowering efficacy.

### Access to the clinical trial data

There are no contractual agreements in place that limit free access to the clinical trial data for the study investigators or any other future collaborators.

### Study oversight and management

#### The study executive committee

The study executive committee comprises Professors Morris Brown, Tom MacDonald and Bryan Williams. The executive committee will be responsible for high-level decisions affecting the running of the trial (eg, closure of study sites, response to emerging safety issues), and will convene quarterly or more frequently, as required.

#### The study steering committee

The study steering committee comprises the Executive Committee and other PI, the study coordinator and representatives from the Data Monitoring Centre. The steering committee will be responsible for setting up, evaluating and reporting the results of the trial. The Steering Committee will convene on an annual basis or more frequently, as required.

## Discussion

The PATHWAY 2 study is the first RCT comparing different drug treatment options in a well-characterised population of patients with resistant hypertension. The study will define whether a specific type of drug is usually dominant with regard to BP lowering efficacy, or whether the response to treatment is more heterogenous. The PATHWAY 2 study also includes assessments of plasma renin and detailed cardiovascular physiological phenotpying, which will provide important insights into underlying mechanisms for resistant hypertension and help determine whether treatment decisions could be better stratified using measurements such as plasma renin. In the absence of any prior RCTs of this type, the data provided by this study will undoubtedly influence future treatment guidelines for the treatment of resistant hypertension.

The PATHWAY 2 study forms part of a trilogy of studies (PATHWAY 1,2,3)^15^ ^16^ currently being undertaken by the British Hypertension Society research network that have been specifically designed by clinical researchers, who are leading regional specialist hypertension clinical services, to tackle fundamental unresolved questions regarding the treatment of hypertension. The need for clinical trial data to inform future recommendations for the treatment of resistant hypertension was also highlighted by NICE as a key research question.^5^

### Challenges in studying resistant hypertension

In the past, studies of resistant hypertension have been difficult because of a lack of consensus with regard to the definition of resistant hypertension and the need to exclude a variety of factors that can lead to apparent or pseudo-resistant hypertension, including (1) poor BP measurement techniques and failure to exclude a so called ‘white coat effect’ in which the clinic BP is elevated despite multiple drug treatments, but BP measured using ambulatory or home BP measurements shows BP to be controlled;^17^ (2) failure to exclude underlying and potentially remediable secondary causes of hypertension, such as aldosterone producing adenomas, renovascular disease, phaeochromocytoma, etc and (3) poor recognition of the scale of non-partial or partial patient concordance with their drug therapy.^1^ ^14^ Furthermore, there has been a longstanding lack of enthusiasm by the big pharmaceutical companies for drug development and clinical trials in this area, in part due to an aversion to developing drug new therapies that would only be used as a fourth-line treatment, as well as the aforementioned lack of consensus regarding the definition of resistant hypertension and a clear understanding of the underlying pathophysiological mechanisms. That said, the recent emergence device-based therapies that have specifically targeted resistant hypertension have served to elevate the profile of resistant hypertension and have also underscored the challenges and complexity in identifying, characterising and studying these patients.^18^ ^19^

### International context for the study

The PATHWAY 2 study has benefited from an emerging international consensus regarding the definition of resistant hypertension,^2^ ^3^ which is consistent with the definition used in our study, based on NICE guidelines.^4^ ^5^ Moreover, all current international guidelines have recently converged and now recommend A+C+D as the preferred three-drug combination at step 3 of their treatment algorithms, which forms the basis of the diagnosis of resistant hypertension.^2–4^ ^20–22^ This means that the findings of our study will have broad international relevance and applicability. Our study also benefits from inclusion of robust methods to monitor treatment concordance and the inclusion of home blood pressure monitoring to exclude a ‘white coat effect’ and provide a more comprehensive assessment of the BP response to the various treatments. In addition, most of our study sites are specialist hypertension centres, ensuring that potential secondary causes for resistant hypertension will have been excluded. Taken together with the renin profiling and detailed physiological phenotyping, the PATHWAY 2 study will be recruiting the most precisely defined resistant-hypertension population to date.

### Choice of drug therapies to be tested

When designing the PATHWAY 2 trial, the potential drug treatment options to be tested were limited by the fact that most patients will already have been treated with A+C+D at baseline. Thus, the potential fourth-line drugs to be tested included further diuretic therapy and sympathetic nervous system (SNS) blockade with either α-blockade or β-blockade. We considered other possible treatments, such as centrally acting drugs, but as these predominantly act through modulation of the SNS, the use of α-blockade or β-blockade would allow us to test whether increased activity of the SNS is a key underlying mechanism for resistant hypertension. This has become even more relevant with the emergence of device-based treatment strategies, such as renal denervation, which are designed to target the SNS.^18^ The choice of spironolactone as ‘further diuretic’ therapy for our study was supported by data, predominantly from observational studies, suggesting that this treatment may be particularly effective in patients with resistant hypertension.^9–11^ This decision was subsequently supported by the NICE guideline recommendation in 2011, which noted that the best available data supported the use of low dose spironolactone for the treatment of resistant hypertension, but noted the absence of RCTs to test this hypothesis and recommended that RCTs were required to better define optimal treatment for resistant hypertension.^4^ ^5^ The testing of an α-blocker as treatment for resistant hypertension was supported by observational data, albeit on a background of two rather than three drugs, suggesting that an α-blocker could be an effective add-on therapy.^23^ The choice of a β-blocker was also logical because in addition to providing an alternative mechanism to inhibit the SNS, β-blockade also suppresses plasma renin and thus allows our ‘α, β, Δ’ rule and plasma renin hypothesis to be tested (see below), which states that a β-blocker would be the most effective treatment when plasma renin is elevated at baseline.

### Evaluating strategies for treatment stratification

The PATHWAY 2 study also incorporates measurement of plasma renin and a detailed physiological evaluation of patients with resistant hypertension (eg, cardiac output, systemic vascular resistance, arterial stiffness and thoracic bioimpedance) to determine if these tests can better predict the best response to a specific drug type. The principal secondary hypothesis tests an ‘α, β, Δ’ rule^8^ which states that (1) further diuretic therapy (Δ), that is, spironolactone, will be the best therapy in patients with evidence of sodium and volume overload, and this will be predicted by a low plasma renin at baseline, despite treatment with A+C+D; (2) that a β-blocker (β), that is, bisoprolol, which suppresses renin would be the best treatment in patients with a high plasma renin at baseline; and (3) that an α-blocker (α), that is, doxazosin, would be the best treatment in those with a plasma renin level in the mid-tertile. Such a finding would suggest potential utility in measuring plasma renin to stratify the most appropriate treatment for individual patients. This concept was suggested many years ago^24^ and previous studies have suggested that physiological phenotyping of sodium/volume status (for which plasma renin is a surrogate) have suggested that this can predict the most effective treatment.^25^ In this regard, our primary hypothesis states that we expect the most common underlying pathophysiology for resistant hypertension will be sodium/volume overload, resulting in a low plasma renin at baseline, and consequently the best response will be obtained with further diuretic therapy, that is, spironolactone, in most patients.

### Evaluating further diuretic therapy

Since spironolactone has been associated with the development of gynaecomastia, estimated to occur in approximately 6% of patients,^9^ we also incorporated an open label run-out phase for the study during which patients will be treated with amiloride, a potassium-sparing diuretic, to determine if this replicates the effects of spironolactone on BP, thus providing an alternative further diuretic therapy for those intolerant of spironolactone.

### Differential BP thresholds for patients with and without diabetes

When this study was designed in 2008, international guidelines had recommended differential BP threshold and targets for people with and without diabetes. Thus, a clinic systolic BP threshold of ≥140 mm Hg was adopted for the definition of uncontrolled BP despite treatment with A+C+D for people without diabetes and ≥130 mm Hg for those with diabetes, with the goal of achieving systolic BP levels below these values. Recent guidelines have adopted a more conservative systolic BP threshold of ≥140 mm Hg for people with diabetes;^2^ ^19^ however, because our study was already well underway by then, we decided not to adjust our inclusion criteria for people with diabetes.

### Double blind versus open label design for the treatment cycles

We designed this study as a double blind study for the treatment cycles, with active study drugs and placebo being identically encapsulated for blinding. The costs and logistics of doing this are substantial and as patients were destined to receive all treatments in random rotation, we questioned the value of the blinding process as the trial progressed. We decided to evaluate the importance of blinding the treatment allocations by adopting an open label design for the final 100 patients recruited into the trial by dispensing the active treatments in their usual packaging. We will conduct analyses to determine if this had any impact on the outcome in the blinded versus unblinded phases of the trial. These findings will provide valuable information with regard to the necessity of the expensive treatment blinding procedures for future studies of this kind.

## Conclusions

The PATHWAY 2 trial is fully recruited and is due to complete in 2015. The dropout rate is within expectations and thus, the trial should be fully powered to test its primary and secondary hypotheses. PATHWAY 2 is the first robust RCT to evaluate the optimal treatment/s for resistant hypertension and will define whether treatment can be best stratified according to plasma renin levels. Whatever the outcome, the findings of PATHWAY 2 are likely to influence future international guidelines for the drug treatment of resistant hypertension and provide insights into underlying mechanisms that could facilitate the targeted development of new therapies for this condition.
